# Botulinum neurotoxin type A for the treatment of pain: not just in migraine and trigeminal neuralgia

**DOI:** 10.1186/s10194-017-0744-z

**Published:** 2017-03-21

**Authors:** Giorgio Sandrini, Roberto De Icco, Cristina Tassorelli, Nicola Smania, Stefano Tamburin

**Affiliations:** 1 C. Mondino National Institute of Neurology Foundation, IRCCS, Pavia, Italy; 2 Department of Brain and Behavioural Sciences, University of Pavia, Pavia, Italy; 3 Department of Neurosciences, Biomedicine and Movement Sciences, University of Verona, Piazzale Scuro 10, I-37134 Verona, Italy; 4 Neuromotor and Cognitive Rehabilitation Research Centre, University of Verona, Verona, Italy

**Keywords:** Botulinum neurotoxin, Primary headaches, Migraine, Neuropathic pain, Pain, Trigeminal neuralgia, Treatment

## Abstract

**Background:**

Despite their huge epidemiological impact, primary headaches, trigeminal neuralgia and other chronic pain conditions still receive suboptimal medical approach, even in developed countries. The limited efficacy of current pain-killers and prophylactic treatments stands among the main reasons for this phenomenon. Botulinum neurotoxin (BoNT) represents a well-established and licensed treatment for chronic migraine, but also an emerging treatment for other types of primary headache, trigeminal neuralgia, neuropathic pain, and an increasing number of pain conditions.

**Methods:**

We searched and critically reviewed evidence for the efficacy of BoNT for the treatment of chronic pain.

**Results:**

Meta-analyses and randomized controlled trials (RCTs) suggest that BoNT potentially represents a multi-purpose drug for the treatment of pain in several disorders due to a favorable safety profile and a long-lasting relief after a single injection.

**Conclusions:**

BoNT is an emerging treatment in different pain conditions. Future RCTs should explore the use of BoNT injection therapy combined with systemic drugs and/or physical therapies as new pain treatment strategies.

## Review

### Introduction

The prevalence of moderate-to-severe chronic non-cancer pain in Europe is estimated to be 20% [1]. A large population-based study on the prevalence of primary headache (PH) disorders in Germany estimated that 2.6% of the general population suffer from headache ≥15 days/month, and 1.1% from chronic migraine [2]. Medication-overuse headache (MOH), i.e., chronic headache resulting from excessive consumption of pain-killers for headache, has an estimated worldwide prevalence of 0.5–7.2% [3]. Around 7% in the general population suffer from some form of chronic neuropathic pain (NP) [4].

The social and economic burden of chronic pain is staggering, in that it interferes with everyday activities, lowers productivity, affects personal relationships, and results in depressive symptoms. WHO ranks migraine as the sixth highest cause of disability worldwide when considered alone, and the third highest when MOH is included [5]. According to U.S. figures, the total annual median cost for medical services and analgesics for the relief of migraine, low back pain, and fibromyalgia is roughly $5,000 and more than $25,000 for treating HIV-related pain and multiple sclerosis [6]. Though lower than the estimated $50,000 spent for ameliorating cancer pain [6], the toll that persistent pain and PH take on overall wellbeing is heavier because of the chronic course of the underlying condition.

Most patients with PH and chronic pain do not receive satisfactory treatment. A European survey reported that 43–81% of patients with headache were not satisfied with their treatment, and the main reason was poor effectiveness of prescribed drugs [7]. Around 25–50% of patients with trigeminal neuralgia (TN) become refractory to drug therapy, and surgical procedures are not always feasible in these patients, and they may occasionally result in severe complications [8]. Only 30–40% of patients with NP achieve a ≥50% pain reduction with currently available therapies [9]. In a recent meta-analysis that took into account publication bias for negative studies, the Special Interest Group on Neuropathic Pain (NeuPSIG) of the International Association for the Study of Pain found that the number-needed-to-treat (NNT) for first-line NP drugs is 3.6–7.7, indicating that less than 30% of patients are responders, and the figures for other treatments are even worse [10]. The number-needed-to-harm (NNH) for these drugs ranges from 11.8 to 31.9, resulting in high drop-out rates [10]. Non-opioid analgesics, nonsteroidal anti-inflammatory drugs, cyclooxygenase-2 inhibitors, and opioids are widely used for relieving nociceptive pain; however, following increased reports of adverse events and side effects, recent warnings and guidelines have been issued, advising caution when prescribing them [11]. Because current pharmacological approaches to PH, TN, NP and other types of chronic pain are inadequate and subject to unintentional abuse, alternative treatment options need to be reconsidered.

Botulinum neurotoxin (BoNT), a potent natural toxin produced by the anaerobic bacterium *Clostridium botulinum*, blocks the release of acetylcholine at the neuromuscular junction by inhibiting the soluble N-ethylmaleimide-sensitive factor attachment protein receptor (SNARE) complex. Since the use of BoNT for the treatment of strabismus was pioneered more than 40 years ago, the therapeutic indications for BoNT type A (BoNT-A) and, more recently, type B for treating excessive and/or undesired muscle tone, have progressively expanded [12]. Currently available BoNT formulations, i.e., abobotulinumtoxin-A (Dysport; Ipsen, Paris, France), incobotulinumtoxin-A (Xeomin, Merz Pharmaceuticals GmbH, Frankfurt, Germany), onabotulinumtoxin-A (Botox; Allergan, Inc., Irvine, CA, USA), and rimabotulinumtoxin-B (Myobloc/Neurobloc; Solstice Neurosciences, Inc., San Francisco, CA, USA) are licensed for the treatment of spasticity and dystonia, and were applied in 17 million treatments between 1994 and 2013 in the United States alone [12]. Though the formulations differ in pharmacological profile, potency, dosage, and approved indications, BoNT is recognized as a safe and effective treatment for spasticity resulting from stroke, multiple sclerosis and spinal cord injury, as well as for dystonia, tremor, and other movement disorders [12]. Guidelines and expert opinions derived from broad-based, high-quality evidence recommend the use of BoNT either alone or in combination with rehabilitation procedures as first-line treatment for spasticity and focal dystonia [13–15].

**Fig. 1:**
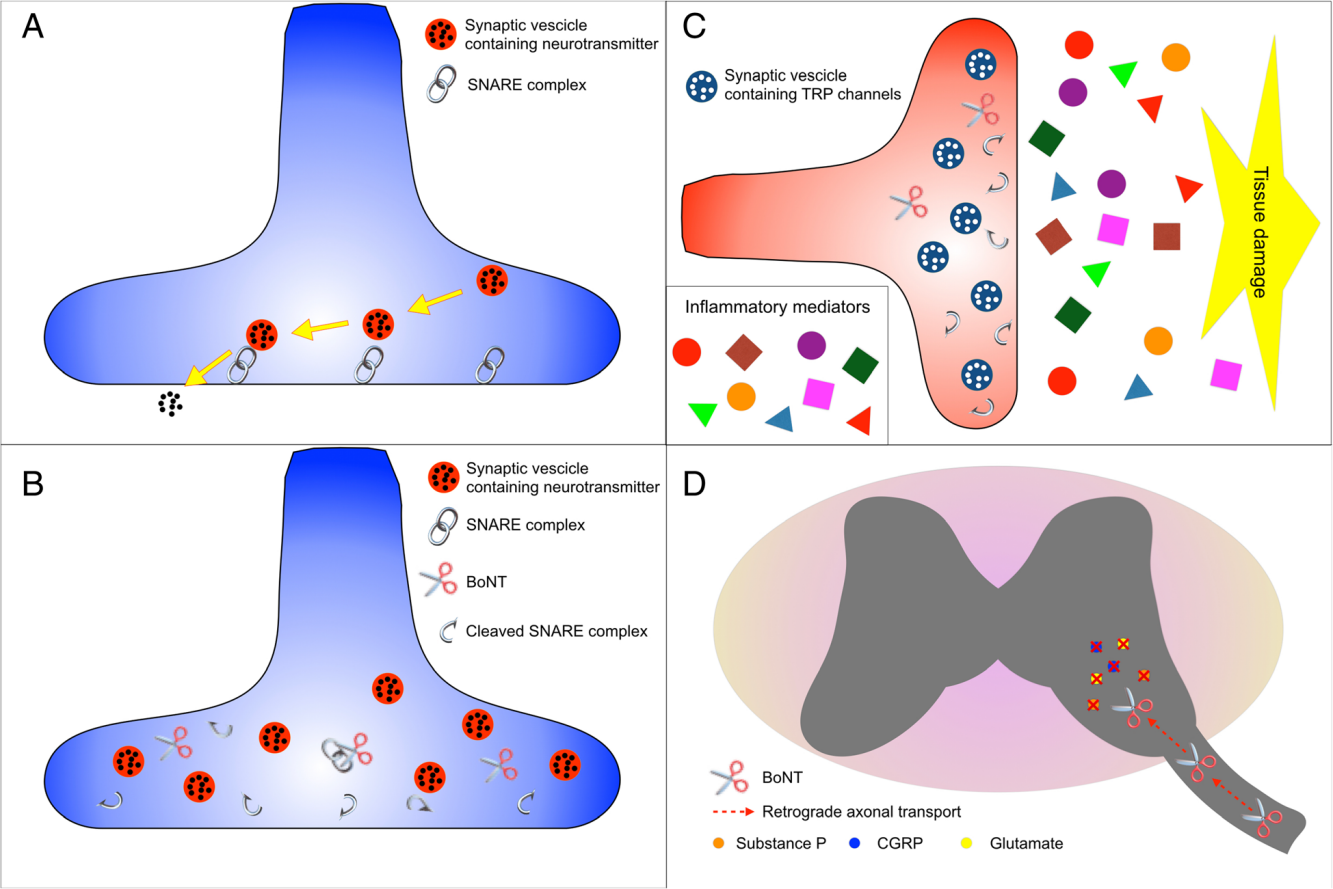
The neurobiological mechanisms of the effect of botulinum neurotoxin (BonT) on pain according to animal models [16] and the anatomical levels where they may take place. Panel **a** shows a normal axon and the role of the soluble N-ethylmaleimide-sensitive factor attachment protein receptor (SNARE) complex, here represented by a chain, for allowing the fusion between the synaptic vescicles (*red circles*) containing a neurotransmitter (*black dots*) and the axonal membrane resulting in the neurotransmitter release. Panel **b** shows the effect of the BoNT, represented by scissors that cleave the SNARE complex and impede vescicle fusion and neurotransmitter release. Panel **c** shows peripheral sensitization after tissue injury, which results in the release of a number of inflammatory mediators (e.g., histamine, bradykinin, prostaglandins, interleukins, adenosine, and nerve growth factors) that, in turn, induce the expression of transient receptor potential (TRP) channels and cause sensitization of the peripheral nociceptor. BoNT may cleave the SNARE complex, block fusion of the vescicles (*blue circles*) containing TRP channels (*white dots*) and reduce peripheral nociceptor sensitization. This mechanism may contribute to the effect of BoNT on nociceptive pain and peripheral neuropathic pain (NP). Panel **d** shows retrograde axonal transport of BoNT to the dorsal horn of the spinal cord where it can block the release of pain-modulating neurotransmitters, such as glutamate, substance P, and calcitonin gene-related peptide (CGRP). This mechanism may reduce central sensitization phenomena and spinal NP

Not surprisingly, BoNT was found to reduce pain in spasticity, dystonia, and related conditions where pain reduction is an important outcome [13, 14]. Serendipitous clinical observations that pain symptoms may improve independently of muscle hyperactivity and with a different time course after BoNT injection have spurred the exploration of the mechanisms underlying this effect in animal models and the collection of evidence in clinical settings [16]. Animal models indicate that BoNT may be effective in controlling pain via its interaction with the SNARE complex that blocks synaptic vesicle fusion and inhibits the release of various pain-modulating neurotransmitters, including glutamate, substance P, calcitonin gene-related peptide, and pain-sensing transmembrane receptors, such as transient receptor potential channels on the neuronal plasma membrane [16]. In addition, growing evidence suggests that the analgesic and anti-inflammatory effects of BoNT are mediated through various molecular pathways in both the peripheral nerves and the spinal cord [16]. Figure 1 summarizes the neurobiological mechanisms through which BoNT may modulate pain, and their possible anatomical levels.

Here we reviewed published evidence on the use of BoNT for the treatment of pain. Since a number of meta-analyses have already been published on this topic, we chose a narrative approach, focusing more specifically on a critical review of current data and possible future approaches.

## Methods

The electronic database MEDLINE (accessed by Pubmed; 1 January 2006–31 December 2016) was searched with the string *((“pain” [MeSH Terms] OR “pain” [All Fields]) AND (“botulinum toxins” [MeSH Terms] OR (“botulinum” [All Fields] AND “toxins” [All Fields]) OR “botulinum toxins” [All Fields] OR (“botulinum” [All Fields] AND “toxin” [All Fields]) OR “botulinum toxin” [All Fields])) AND (Clinical Trial[ptyp] OR (“meta-analysis” [Publication Type] OR “meta-analysis as topic” [MeSH Terms] OR “meta-analysis” [All Fields]))* with no language restrictions and all the titles and abstracts identified by the search were evaluated for eligibility.

## Results

The PubMed searched yielded 84 randomized controlled trials (RCTs) and 36 meta-analyses or systematic reviews, indicating a consistent bulk of data on the role of BoNT-A for the treatment of chronic pain. The conditions for which at least one meta-analysis was available are summarized in Table 1.

**Table 1:** Evidence for the use of BoNT-A in chronic pain conditions

Condition	Subjects	Studies	Comparator	Outcome^a^	Ref.
EM	1838	9	Placebo	n.s.	18
CM	1508	5	Placebo	HEPM: −2.30 [95% CI: −3.7, −0.9]	18
	59	1	Topiramate	n.s.	18
	72	1	Amitriptyline	n.s.	18
CDH	1115	1	Placebo	HEPM: −2.1 [95% CI: −3.6, −0.6]	18
Any TTH	59	1	Valproate	n.s.	18
	21	1	Steroids	HEPM: −2.5 [95% CI:−3.5, −1.5]	18
Chronic TTH	675	7	Placebo	n.s.	18
TMD	145	4	Placebo	Meta-analysis not performed	22
	30	1	Manipulation	n.s.	22
CNP^b^	371	8	Placebo	Little or no difference	23
CCH	32	1	Placebo	Little or no difference	23
Whiplash	96	3	Placebo	n.s.	24
TN	178	4	Placebo	PPD: −29.8 [95% CI:−38.5, −21.1]	8
Diabetic NP	76	2	Placebo	0–10 VAS: −2.0 [95% CI:−3.1, −0.8]	27
Peripheral NP	68	1	Placebo	0–10 NRS:-0.8 [95% CI:−1.0, −0.6]	26
Spinal NP	40	1	Placebo	Significant VAS reduction	28
Myofascial	332	8	Placebo	n.s.	24
PSSP	86	5	Placebo	0–10 VAS: −1.2 [95% CI:−2.4, −0.1]	29
ASP	40	1	Placebo	0–10 VAS −2.0 [95% CI:−3.7, −0.3]	29
LE	274	4	Placebo	ES: −0.5 [95% CI:−0.9, −0.1]	31
LBP	131	3	Mixed^c^	Meta-analysis not performed	33
Ankle OA	75	1	HA	n.s.	34
PF	133	3	Placebo	n.s.	35
	136	2	Steroids	Pain relief: −0.7 [95% CI:−1.0, −0.3]	35
BPS	317	6	Placebo	0–10 VAS −1.7 [95% CI:−3.2, −0.3]	38

Here are reported chronic pain conditions for which at least one meta-analysis or systematic review was available. *ASP* Arthritic shoulder pain, *BoNT-A* Botulinum neurotoxin type A, *BPS* Bladder pain syndrome, *CCH* Chronic cervicogenic headache, *CDH* Chronic daily headache, *CI* Confidence interval, *CM* Chronic migraine, *CNP* Chronic neck pain, *EM* Episodic migraine, *ES* Effect size, *HA* Hyaluronic acid, *HEPM* Headache episodes per months, *LBP* Low back pain, *LE* Lateral epycondylitis, *NP* Neuropathic pain, *NRS* Numerical rating scale, *n.s.* Not significant, *OA* Osteoarthritis, *PF* Plantar fasciitis, *PPD* Paroxysms per day, *PSSP* Post-stroke shoulder pain, *TMD* Temporomandibular disorders, *TN* Trigeminal neuralgia, *TTH* Tension type headache, *VAS* Visual analogue scale

^a^Results of the comparison between BoNT and comparator

^b^Physiotherapeutic exercise and analgesics were combined with both BoNT and placebo in two studies (*n* = 95 patients)

^c^Placebo, acupuncture or steroids

Based on the PREEMPT program that included data from two multicenter RCTs (*n* = 1384 patients), BoNT-A was given regulatory approval for the treatment of chronic migraine (CM) in 2010, the fact notwithstanding that its mechanism of action is not yet completely elucidated [17]. A meta-analysis on the role of BoNT as a prophylactic treatment of migraine showed that BoNT-A compared with placebo was associated with a small-to-modest benefit for chronic daily headache and CM (i.e., a mean −2.1 to −2.3 reduction in headache episodes per months), but was not associated with fewer episodic migraine or chronic tension-type headaches (TTHs) per month [18]. A RCT on the treatment of MOH failed to document an effect on the headache days, but showed a reduction of drug consumption [19]. Overall, BoNT adverse events were few and not serious in CM patients [20].

Open-label data suggest that intramuscular injection of BoNT-A in the masseter muscle may improve pain in patients with temporomandibular disorders (TMD) and TTH [21]. Systematic reviews on TMD reported significant myofascial pain reduction following BoNT treatment in comparison to placebo in two RCTs, no significant difference in two RCTs, and equal efficacy of BoNT and fascial manipulation in one RCT, but a meta-analysis was not possible because of considerable variations in study design and outcomes [22].

Five high quality RCTs indicated little or no effect of BoNT-A, and two very low quality RCTs suggested little or no difference between BoNT-A and placebo, both combined with physiotherapeutic exercise and analgesics, in patients with chronic neck pain [23]. A very low quality RCT showed little or no effect of BoNT-A in chronic cervicogenic headache [23]. Three RCTs showed no effect of BoNT-A in pain related to whiplash injury [24].

A recently published meta-analysis concluded that BoNT-A may be an effective and safe treatment option for patients with TN, in that it yielded, on average, a −29.8 reduction in paroxysms per day [8]. Conversely, BoNT was not found to be effective in occipital neuralgia [25].

RCTs have documented that BoNT-A may be effective in peripheral NP, including painful diabetic neuropathy, post-herpetic and post-traumatic neuralgia [26]. A meta-analysis including two studies on diabetic NP showed a −2.0 reduction on a 0–10 visual analogue scale (VAS) following treatment with BoNT-A, resulting in clinically significant improvement of minimum change in pain, with no more adverse effects than placebo [27]. A RCT on peripheral NP documented a −0.8 reduction in numerical rating scale over 24 weeks compared with placebo [26]. A RCT on NP secondary to spinal cord injury showed significant reduction at 4 weeks (active: 18.6 ± 16.8; placebo: 2.6 ± 14.6) and 8 weeks (active: 21.3 ± 26.8; placebo: 0.3 ± 19.5) after BoNT-A [28]. The NeuPSIG recommended BoNT-A as third line pharmacological treatment for NP, assigning it the same strength of recommendation as strong opioids [10]. This recommendation was largely based on the high tolerability of BoNT-A and the safety concerns related to opioids, despite the fact that effectiveness and the bulk of evidence favoring opioids, particularly oxycodone and morphine, is far more robust [10].

Eight RCTs documented no significant effect of BoNT-A in myofascial pain syndrome [24].

Studies with a small sample size and a high risk of bias have suggested that intramuscular BoNT-A injection may reduce pain (i.e., on average between −1.2 and −2.0 points on a 0–10 VAS) and improve function in chronic shoulder pain from spastic hemiplegia or arthritis [29]. A similar effect was documented after intra-articular BoNT-A injection for refractory hemiplegic shoulder pain [30].

Four RCTs documented a moderate effect on pain to BoNT-A 60 U injection into the forearm extensor muscles in chronic treatment-resistant lateral epicondylitis [31]. A recent Bayesian network meta-analysis that compared different injection therapies for lateral epicondylitis showed a not significant trend towards better effect than placebo for BoNT-A [32].

Three RCTs showed that BoNT-A improves pain, function, or both better than saline injections, acupuncture or steroid injections in patients with low back pain and sciatica, but the low-quality and heterogeneity of studies impeded a meta-analysis [33].

A RCT on ankle osteoarthritis reported no significant difference between BoNT and hyaluronic acid, which in turn appeared not more effective than exercise therapy, and did not seem to offer clinically relevant advantage in comparison to placebo [34]. Data from three RCTs documented no significant difference between BoNT-A and placebo, and those from two RCTs showed slight significant advantage of BoNT-A over corticosteroids in plantar fasciitis, but a network meta-analysys including 22 RCTs indicated BoNT-A as the most likely treatment to relieve pain in this condition [35].

Limited evidence of efficacy for peripheral BoNT injection therapy for residual limb pain after amputation is suggested by very small trials and case series, while a small RCT showed that BoNT-A was not superior to lidocaine and methylprednisolone in this condition [36].

Pelvic pain, also known as bladder pain syndrome (BPS) or interstitial cystitis, is a complex condition with an ill-defined pathogenesis [37]. Six RCTs showed that intravesical BoNT-A injections might offer significant improvement in pain (i.e., average −1.7 points reduction on a 0–10 VAS), daytime urination frequency, and maximum cystometric capacity for patients with refractory BPS [38].

BoNT-A was found to improve post-surgical and post-radiation pain in cancer patients in a small open-label study [39].

## Discussion and conclusions

The current evidence reviewed here indicate that BoNT-A represents an effective prophylactic treatment for CM, and may be helpful in other types of cranial, facial, and cervical pain, as well as in patients with TN, peripheral and spinal NP, some types of pain secondary to musculoskeletal diseases, and BPS. For most of these conditions, however, the quality of evidence is low, and the sample size of RCTs is small. In some conditions, such as NP, where the evidence for first-line treatments (i.e., α_2_-δ ligands, antidepressants) comes from a consistently larger number of patients, evidence supporting the use of BoNT should be considered with caution and preliminary.

Neurobiological grounds for the use of BoNT in pain come from experimental models [16]. Despite the heterogeneity of conditions, routes of administration, and outcomes, studies that sought to identify best-responder profiles suggest that complete denervation or nerve transection predicts poor pain response to BoNT [26, 28]. This clinical observation is in keeping with animal models that indicate that the analgesic effect of BoNT is mediated, at least in part, by retrograde axonal transport to the spinal cord [16].

According to the International Classification of Headache Disorders, the essential diagnostic criteria of PH disorders are based on the presence of specific clinical features and the absence of decisive pathological or radiological findings. It is noteworthy that the classification of a patient in a given PH subtype may change in a quarter of cases, especially when the diagnosis is probable, even in expert centers [40]. Patients suffering from chronic migraine frequently report other types of pain (i.e., fibromyalgia), and both types of pain are usually more or less refractory to common treatments [41].

Identifying different types of pain (e.g., neuropathic, osteoarticular, or associated with excessive muscle tone) in the same patient represents the first step towards developing a more appropriate and mechanisms-based pharmacological prescription for pain [11]. Guidelines and diagnostic algorithms for the management of NP may be difficult to apply in clinical conditions such as low back pain, for example, where different types of pain coexist [42]. While stratification of NP patients by their underlying sensory profile has been suggested to better inform the design of RCTs and personalized pharmacological treatment, the clinical utility of this tempting approach has not yielded more favorable NNT-NNH profiles for NP drugs [9]. Clinical scenarios are more complex and ‘real-world’ patients are very different from those enrolled in RCTs. Diagnosis of NP according to the NeuPSIG diagnostic algorithm has many merits, including different levels of certainty (i.e., probable, possible, definite), but it requires a solid knowledge of central and peripheral nervous system anatomy that not all pain physicians may possess [42]. The elderly and neurological patients may have several comorbidities and cognitive impairments that can blur the clinical expression of pain. Furthermore, thorough examination to evaluate the different types of pain may not be possible due to time constrictions in some cases.

BoNT-A was found to be effective for relieving PH, TN, NP, nociceptive and osteoarticular pain, as well as pain symptoms associated with muscle hypertonus. Its effect on different pain mechanisms coexisting in the same patient, together with its favorable side effect profile and long-lasting pain relief after a single injection, when effective, make BoNT-A a potential multi-purpose drug for pain treatment in a variety of neurological and non-neurological conditions. There are, however, several open questions that warrant further studies.

PREEMPT data showed a mean reduction of approximately 2 headache days out of 4 weeks after BoNT-A in comparison to placebo in chronic migraine patients [17]. Similarly, in many RCTs, self-rated pain reduction after BoNT-A injection was generally no more than 2 points on a visual analogue or numerical rating scale from 0 to 10 [26, 28, 29], which is insufficient to document a satisfactory response in most patients. The association of an ad hoc rehabilitative program was found to improve the clinical efficacy of BoNT in cervical dystonia [43]. A future area of focus for RCTs should be to explore the potential role of BoNT injection therapy in combination with systemic drugs and/or physical therapies.

Furthermore, BoNT was found to be effective on pain through various routes of injection, including subcutaneous, intramuscular, and intraarticular ones. A small RCT showed that BoNT-A profoundly prolonged (i.e., 2 months on average) analgesia after bupivacaine sympathetic blocks in patients with complex regional pain syndrome, a devastating pain conditions with no established treatment [44]. Comparison of different routes of injection might offer new pieces of information for optimizing the use of BoNT for the treatment of pain.

The most appropriate BoNT dosage is still unknown. In MOH, onabotulinumtoxin-A 100 U was found to be ineffective on the headache days in a RCT [19], while an open label prospective study that evaluated two doses of onabotulinumtoxin-A (i.e., 155 U and 195 U) reported a significant reduction of the number of headache and migraine days for both doses, but with a superiority of the higher dosage [45]. These data suggest that dose-finding studies may represent another promising field of research.

## Abbreviations

BoNT: Botulinum neurotoxin
BoNT-A: Botulinum neurotoxin type A
CM: Chronic migraine
MOH: Medication-overuse headache
NeuPSIG: Special Interest Group on Neuropathic Pain
NNH: Number-needed-to-harm
NNT: Number-needed-to-treat
NP: Neuropathic pain
PH: Primary headache
RCT: Randomized controlled trial
SNARE: Soluble N-ethylmaleimide-sensitive factor attachment protein receptor
TMD: Temporomandibular disorders
TN: Trigeminal neuralgia
TTH: Tension-type headache
